# Association between Chinese youth’s sources of sexual knowledge and sexual and reproductive health: a mediation analysis of sexual knowledge level

**DOI:** 10.1080/26410397.2025.2517430

**Published:** 2025-08-04

**Authors:** Xinye Zou, Kefan Xue, Siyu Zou, Angela Y. Xiao, Wenzhen Cao, Kun Tang

**Affiliations:** a PhD Candidate, School of Humanities and Social Sciences, University of Cambridge, Cambridge, UK; b Research Assistant, Vanke School of Public Health, Tsinghua University, Beijing, People’s Republic of China; c PhD Candidate, Oxford School of Global and Area Studies, University of Oxford, Oxford, UK; d Master of Science Candidate, Department of Epidemiology, Johns Hopkins Bloomberg School of Public Health, Baltimore, MD, USA; e Graduate Student, Department of International Studies, Macalester College, Saint Paul, MN, USA; f Lecturer, School of Public Health and Management, Wenzhou Medical University, Chashan University Town, Wenzhou, People’s Republic of China; g Associate Professor, Vanke School of Public Health, Tsinghua University, Beijing, People’s Republic of China.

**Keywords:** sexual knowledge, sexual and reproductive health, Chinese youth, mediation analysis, sexuality education

## Abstract

Changing sexual behaviours among Chinese youth have emerged along with China’s rapid socioeconomic transition. In the context of enduring Confucian traditions and limited formal sexuality education, there is an increasing demand from Chinese youth for accessible and inclusive sexuality education. This research explores how young people in China seek out and engage with sexual knowledge within this shifting cultural landscape. While previous studies indicate that a lack of knowledge in sexual and reproductive health (SRH) is associated with risky sexual behaviours, investigations on how Chinese youth access SRH knowledge and its impact on their behaviours remain unclear. Based on the survey data collected in 2019–20, this cross-sectional study of 52,256 Chinese youth highlighted how young people access and apply sexual knowledge. Boys were more likely to learn from media (primarily pornography), while girls tended to rely on school-based education and parental communication. Mediation analyses were performed to explore the association between sources of sexual knowledge and sexual and reproductive health with a mediator of sexual knowledge scores. The results show that youth who acquired SRH knowledge from the media were more likely to engage in sexual intercourse and experience early sexual debut, while youth gaining SRH knowledge from school were less likely to have sexual intercourse and early debut. Sexual knowledge acquisition occurs not only in one setting but across various settings in young people’s lives. This study provides insights for future sexuality education improvement through different learning sources and emphasises how young people’s learning experiences impact their sexual behaviour.

## Plain language summary

We studied how young people in China learn about sex and how it affects their choices. China is changing fast, so we wanted to see how these changes are shaping young people’s behaviours. We asked 52,256 young people about where they learn about sexual and reproductive health (SRH) and how it influences their actions. We found that boys and girls often learn about SRH from different places. Boys were more likely to learn from the media, primarily pornography. Girls tended to rely on school-based sex education and information from parents. Our study showed that learning from media was linked to starting sexual activity earlier. But young people who learned about SRH at school were less likely to start early. This demonstrates that it matters where young people get their information about sex. Our research shows that young people don’t just learn about sex at home or school – they learn from many places. To help young people make good choices, we need to think about all these different ways they get information when creating sexuality education programs.

## Résumé

L’évolution des comportements sexuels chez les jeunes Chinois a accompagné la rapide transition socio-économiques du pays. Dans un contexte de traditions confucéennes persistantes et d’une éducation à la sexualité formelle limitée, la jeunesse chinoise demande de plus en plus une éducation sexuelle accessible et inclusive. Cette étude explore comment les jeunes en Chine recherchent et utilisent les connaissances sexuelles dans ce paysage culturel en mutation. Si de précédentes études indiquent qu’un manque de connaissances en matière de santé sexuelle et reproductive (SSR) est associé à des comportements sexuels à risque, les investigations sur la manière dont les jeunes Chinois accèdent aux connaissances sur la SSR et son impact sur leurs comportements restent floues. S’appuyant sur les données d’une enquête menée en 2019, cette étude transversale réalisée auprès de 52 256 jeunes Chinois a mis en lumière la manière dont les jeunes accèdent aux connaissances sexuelles et les mettent en pratique. Les garçons étaient plus susceptibles d’apprendre à partir des médias (principalement la pornographie), alors que les filles tendaient à s’appuyer sur l’éducation scolaire et la communication parentale. Des analyses de médiation ont été réalisées pour explorer l’association entre les sources de connaissances sexuelles et la santé sexuelle et reproductive à l’aide d’un médiateur des scores de connaissances sexuelles. Les résultats montrent que les jeunes ayant acquis leurs connaissances en matière de SSR dans les médias avaient plus de probabilités d’avoir des rapports sexuels et d’avoir un premier rapport précoce, tandis que ceux qui avaient obtenu leurs connaissances de SSR à l’école étaient moins susceptibles d’avoir des rapports sexuels et d’avoir un premier rapport précoce. L’acquisition des connaissances sexuelles se produit non pas dans un environnement unique, mais dans différents contextes de la vie des jeunes. Cette étude donne des éclairages pour l’amélioration future de l’éducation à la sexualité par le biais de différentes sources d’apprentissage et souligne l’impact des expériences d’apprentissage des jeunes sur leur comportement sexuel.

## Resumen

Con la rápida transición socioeconómica de China han surgido comportamientos sexuales cambiantes entre las personas jóvenes chinas. En el contexto de tradiciones confucianas duraderas y limitada educación en sexualidad formal, la juventud china exige cada vez más una educación en sexualidad accesible e inclusiva. Esta investigación explora cómo las personas jóvenes en China buscan y aplican el conocimiento sexual en este panorama cultural cambiante. Si bien estudios anteriores indican que la falta de conocimiento de salud sexual y reproductiva (SSR) está asociada con comportamientos sexuales riesgosos, las investigaciones sobre cómo la juventud china obtiene conocimiento de SSR y su impacto en sus comportamientos siguen sin aclararse. Basado en los datos de encuesta recolectados en 2019, este estudio transversal con 52,256 jóvenes chinos destacó cómo las personas jóvenes obtienen y aplican su conocimiento sexual. Los niños eran más propensos a aprender de los medios de comunicación (principalmente pornografía), mientras que las niñas se inclinaban a depender de la educación escolar y la comunicación con sus padres. Se realizaron análisis de mediación para explorar la asociación entre las fuentes de conocimiento sexual y la salud sexual y reproductiva con un mediador de puntajes de conocimiento sexual. Los resultados muestran que las personas jóvenes que adquirieron conocimiento de SSR de los medios de comunicación eran más propensas a tener coito sexual y una iniciación sexual precoz, mientras que la juventud que adquirió su conocimiento de SSR en la escuela era menos propensa a tener coito sexual y una iniciación sexual precoz. Las personas jóvenes adquieren conocimiento sexual no solo en un entorno, sino en diversos entornos de su vida. Este estudio ofrece ideas para mejorar la futura educación en sexualidad mediante diferentes fuentes de aprendizaje y hace hincapié en cómo las experiencias de aprendizaje de las personas jóvenes afectan su comportamiento sexual.

## Introduction

As China undergoes rapid socioeconomic changes, the sexual attitudes and behaviours of its youth have become noticeably more liberal^1^. The prevalence of premarital sexual activity has risen greatly. For example, the average age of first sexual experiences dropped from 22.8 years in 2009 to 18.4 years in 2018.^2,3^ Engaging in sexual activity at a younger age is not inherently negative; rather, in supportive and informed contexts, it can provide opportunities for young people to put into practice safe, thoughtful, and informed decision-making about relationships, sexuality and reproduction.^4^ Nevertheless, without adequate sexual health education and targeted interventions, this trend poses risks to the sexual and reproductive health (SRH) of Chinese youth^5^.

Risky sexual behaviours involve actions that increase the chances of negative health outcomes, such as sexually transmitted infections (STIs) and unintended pregnancies. These behaviours may include starting sexual activity at a young age, having multiple partners, engaging in casual sex, or not using condoms regularly or at all during sexual encounters.^6^ Research evidence has shown a gradual increase in premarital sexual activity, low rates of contraceptive use, and rising rates of HIV and unintended pregnancy among Chinese university students.^7^ For instance, a regional study in Shanghai reported that 13.8% of premaritally sexually active female youth (aged 15–24 years old) had an unintended pregnancy.^8^ Meanwhile, reported cases of HIV/AIDS among Chinese youth surged, rising from 5,186 in 2005 to 10,195 in 2012.^9^ Multiple studies in China have shown both quantitatively and qualitatively that insufficient or poorly implemented SRH education is associated with risky sexual behaviours, such as earlier sexual debut, inconsistent condom use, and limited contraceptive knowledge among youth.^10,11^ In a 2017 survey covering 1,355 youth in 11 Chinese provinces, only 53.28% of participants achieved a score of 60 or above on a scale of 100 on SRH knowledge,^12^ indicating insufficient SRH knowledge among Chinese youth. However, the 1994 International Conference on Population and Development indicated access to SRH information as a basic human right, and SRH is central to women’s empowerment.^13^ The quality of sources from which young people obtain SRH knowledge is thus crucial for their health and development.^14^ A growing number of studies have documented Chinese youth’s changing sexual behaviour^15^; however, studies examining the association between patterns of SRH knowledge learning and Chinese youth’s sexual behaviour remain scarce.^16^ This study adopted the latest national survey on SRH carried out by the China Family Planning Association to analyse this association.

Applying Bernstein’s theoretical framework – which distinguishes formal and informal knowledge acquisition^17^ – to sexual learning suggests that it derives from both formal sources, such as school-based sexuality education (any formal courses on sexual and reproductive health offered in schools) and informal sources, such as parents, the media, and peers. Earlier studies have shown that formal school-based sexuality education, when comprehensive and of high quality, can promote safer sexual behaviours.^18^ In contemporary China, formal school-based sexuality education integrates moral education with public health objectives under state-led initiatives. It primarily adopts an “abstinence-only” framework, emphasising premarital abstinence, personal hygiene, and adherence to societal moral standards. This model is deeply rooted in the notion of fostering self-control and social responsibility among young people,^19^ along with longstanding Chinese traditional Confucianism and Taoism. However, formal school-based SRH education in China remains problematic, with low effectiveness.^7,20^ For example, a previous study suggests that while formal school-based sexuality education in China incorporates elements of scientific education, such as information on reproductive health, it largely avoids discussing topics like safe sex in depth, focusing instead on discouraging premarital sexual behaviour and reinforcing traditional moral values^19^.

In contrast, parent–adolescent communication is an informal SRH learning source that plays a protective role in adolescent sexual socialisation.^21^ In our study, parental communication about sexuality was assessed based on the extent to which parents provided informative or supportive answers to adolescents’ questions. Previous studies in other contexts suggest that higher quality parent–adolescent communication strongly correlates with safer and healthier sexual behaviours among adolescents.^22^ Nevertheless, China-specific research evidence suggests that under traditional sociocultural norms, sex-related communication is usually not openly conducted in the family, or even remains taboo.^23^ In Chinese families, communication of SRH knowledge is usually insufficient, gendered, fear- or consequence-based, and of poor quality^21^.

Therefore, the lag in school-based sexuality education and the lack of parent–adolescent communication have led Chinese adolescents and youth to seek and obtain sexual knowledge from the media. While the media can be a less embarrassing alternative for young people to explore sensitive topics, their accessibility as a source of sexuality education can be both positive and negative. Internationally, digital platforms have proven to be effective tools in promoting sexual health education. For instance, e-learning tools in sub-Saharan Africa have successfully addressed geographical and social barriers to sexual health knowledge.^24^ In the Asia-Pacific region, targeted social media campaigns have raised awareness of contraception and STI prevention among adolescents.^25^ However, these benefits often come with the risk of misinformation, which can negatively influence behaviour and perceptions.^26^ In the Chinese context, media sources such as online platforms, explicit novels, pictures, videos, and social media platforms like QQ, WeChat, and Momo remain central to youth sexuality education but require careful scrutiny to ensure healthy outcomes^27^.

One of the major media sources for young people in China to acquire sexual information is pornography. Pornographic materials, although strictly limited and difficult to access in China, are still prevalent among young people as they offer high privacy and anonymity.^28^ For instance, in a survey of 1,403 university students, almost 60% of participants had consumed pornography, which was higher than the proportion of those who had ever had a contraception-related conversation with their parents (7.1%).^29^ The display of unsafe, highly gendered, and stereotypical sexual practices may potentially affect young people’s healthy sexual development.^30^ Previous studies have demonstrated that pornography use among adolescents is associated with risky sexual behaviours, such as earlier age of sexual debut, condomless sex, multiple sexual partners, and substance use before sex.^31–33^ There are also studies exploring the patterns and outcomes of consuming pornographic materials in LGBTQ adolescents.^34^ However, pornography as a pattern of learning sexual information, and its role in sexual behaviours among Chinese youth, are understudied, with only a few studies demonstrating the association. For example, a study in 2013 covered 19,123 participants across nine provinces to explore risky sexual behaviour and condom use in Chinese universities, demonstrating how the frequency of pornography consumption positively associates with frequency of sexual intercourse.^35^

This study focuses on analysing youth’s SRH knowledge learning patterns in the Chinese sociocultural context, including formal school-based sexuality education and informal learning from parents and media*.* We intend to explore how learning sources are associated with youth sexual behaviour, including sexual intercourse, early debut (<16 years old), casual sex, and condom use during the first intercourse. Additionally, reproductive health outcomes, including STIs and unintended pregnancy, are assessed as part of the broader understanding of young people’s sexual behaviours. The analysis also considers the role of sex and sexual orientation. Literature contributions and substantial implications for researchers, educators, and public health professionals are also discussed.

## Methods

### Study design and participants

We worked with the China Family Planning Association (CFPA) to conduct a large web-based survey between November 2019 and February 2020. A multistage sampling approach was used to ensure a representative distribution of higher education institutions across all 31 provincial-level administrative regions in China, considering geographic location and institution type. The first stage balanced the distribution of higher education institutions across provinces by using administrative divisions provided by the National Bureau of Statistics, categorising geographic location into eastern, central, and western regions. Within each region, institutions were categorised into four levels for undergraduate institutions (first-class universities, first-class academic discipline universities, ordinary undergraduate institutions, and private undergraduate institutions), and three levels for vocational institutions (key vocational colleges, ordinary vocational colleges, and private vocational colleges). A total of 241 colleges and universities were selected with the number of institutions sampled from each category being proportionate to their distribution nationwide. Study participants were recruited using snowball sampling, in which institutional contact persons initially distributed unique web links to the electronic survey questionnaire via social media and institutional networks, encouraging participants to share the link with peers within their academic and social circles. Before starting the electronic survey, participants were shown an informed consent page. Only those who agreed to the terms could proceed to the questionnaire; those who did not consent were automatically exited from the survey.

## Measures

### Sources of sexual learning

Participants were asked whether they had ever “learned about sexual information” from (1) media (yes/no), (2) parents (yes/no), and (3) school (yes/no).

To measure the learning source of media, participants were asked whether they had ever engaged in at least one of the following four actions: (1) actively searched for sexual information including contraception on the Internet; (2) actively searched for pornography online, such as novels, explicit pictures, movies; (3) read or seen pornographic books or magazines; and (4) received pornography on social software, such as QQ, WeChat, and Momo.

Parent–adolescent communication about sex was accessed by asking participants, “When you ask your mother/father about sex, could they answer your questions?” The outcome was coded as 0 if the respondent answered, “Never asked”, “Avoid making any reply”, or “Not only did not answer, but also scolded” (i.e. didn’t have parent–adolescent communication) and 1 if they answered “Sometimes made answers” or “Satisfactorily explained” (i.e. had parent–adolescent communication).

School education was assessed by asking the participant, “Have you attended any sexual and reproductive health education or adolescent education courses at school?” (yes/no).

### SRH knowledge level

Students’ SRH knowledge level was assessed using nine questions (all single-choice questions, coded as SRH knowledge score: 1–9 scores). The quiz contained nine questions on four dimensions: knowledge of contraceptive use, and the results of unsafe sex, such as STIs and unintended pregnancy (see Supplemental Table S1). The response options included “true”, “false”, and “not sure”. Each item was coded as 0 if the respondent answered wrong or “not sure”, and 1 if they answered right. The maximum attainable score was 9 points.

### Sexual orientation

Sexual orientation was measured by asking participant, “Which of the following options do you think best describes your sexual orientation?” The response choices included “heterosexual”, “homosexual”, “bisexual”, “questioning”, and “others”. The variable was coded as 0 if the respondent answered “heterosexual” (i.e. heterosexual) and 1 if they answered “homosexual”, “bisexual”, or “questioning” were coded as 1 (i.e. sexual minority).

### Sexual behaviours

We examined sexual behaviours, including sexual intercourse, early debut, casual sex, and condom use during the first intercourse. Having had sexual intercourse was assessed by asking participants, “Have you ever had sexual intercourse? (By sexual intercourse, we mean penile–vaginal intercourse or penile–anal intercourse)”. The outcome was coded as 1 if the respondent answered “Yes” and 0 if they answered “No”. Participants who had sexual intercourse were asked, “When did you first have sexual intercourse?” Early debut was defined as participants who had sexual debut before the age of 16. Casual sex was measured by asking participants who had sexual intercourse “Have you ever had casual sex? For example, one-night stands, and prostitution”. Condom use during the first intercourse was assessed by asking the participants “Did you use condom during the first intercourse?” The outcome of early debut, casual sex, and condom use during the first intercourse were all coded as 1 if the respondent answered “Yes” and 0 if they answered “No”.

### Reproductive health outcomes

Two reproductive health outcomes were measured in this study, including STIs (yes/no) and unintended pregnancy (yes/no).

### Other covariates

Participants completed a demographic questionnaire collecting information on age (15–24 years old), biological sex (female/male), region (rural/urban), paternal educational achievement (no formal schooling/primary school/middle or high school/college or above), maternal educational achievement (no formal schooling/primary school/middle or high school/college and above), personal expenditure per month (<1000/1000–1499/1500–2000/>2000 CNY), and ethnicity (Han/Minority). All the covariates were treated as categorical variables.

## Ethical statement

The study was reviewed and approved by the Institutional Review Board (IRB) of Tsinghua University (#20190083) on October 30th 2019. Study participants were youth aged 15–24 years, including some minors. While the focus was on university students, the inclusion of different types of institution within the Chinese higher education system encompassed minors enrolled in these institutions. In China, the Personal Information Protection Law (PIPL) treats children's personal information (under 14 years old) as sensitive (Article 28). Parental consent is typically required for processing sensitive information, including research data (Article 31).^36^ However, since our participants were aged 15–24 years, they are legally empowered to give consent to participate and this process was approved by the IRB. Moreover, all participants were reached through official university channels and provided informed consent online before participating. Participation was voluntary, with the option to withdraw at any time. The survey was conducted anonymously, and all identifying information was removed during analysis to ensure confidentiality and ethical compliance. The survey data was collected using Questionnaire Star, the most widely used online survey platform in China that ensures secure data transmission through encryption. The data was stored on encrypted drives with access restricted to authorised personnel, and all analysis was carried out on secure, password-protected computers without any personal identifiers. This research received no funding from any funding agency in the public, commercial or not-for-profit sectors.

## Statistical analysis

First, descriptive statistics were conducted to describe the demographic characteristics, sources of sexual learning, sexual knowledge scores, sexual behaviours, and reproductive health outcomes among heterosexual and sexual minority youth.

Second, a logistic regression analysis was conducted to explore the association between sexual learning sources and sexual behaviours and reproductive outcomes, stratified by sexual orientation and biological sex. All models were adjusted for sexual knowledge, age, region, personal expenditure, parental educational achievement, and ethnicity. Odds ratios (ORs) and 95% confidence intervals (CIs) were calculated.

Third, a linear regression analysis was conducted to explore the association between sexual learning sources and sexual knowledge scores (0–9 scores), stratified by sexual orientation and biological sex. All the models were adjusted for age, region, personal expenditure, parental educational achievement, and ethnicity. The coefficients and 95% confidence intervals (CIs) were reported.

**Figure 1.**
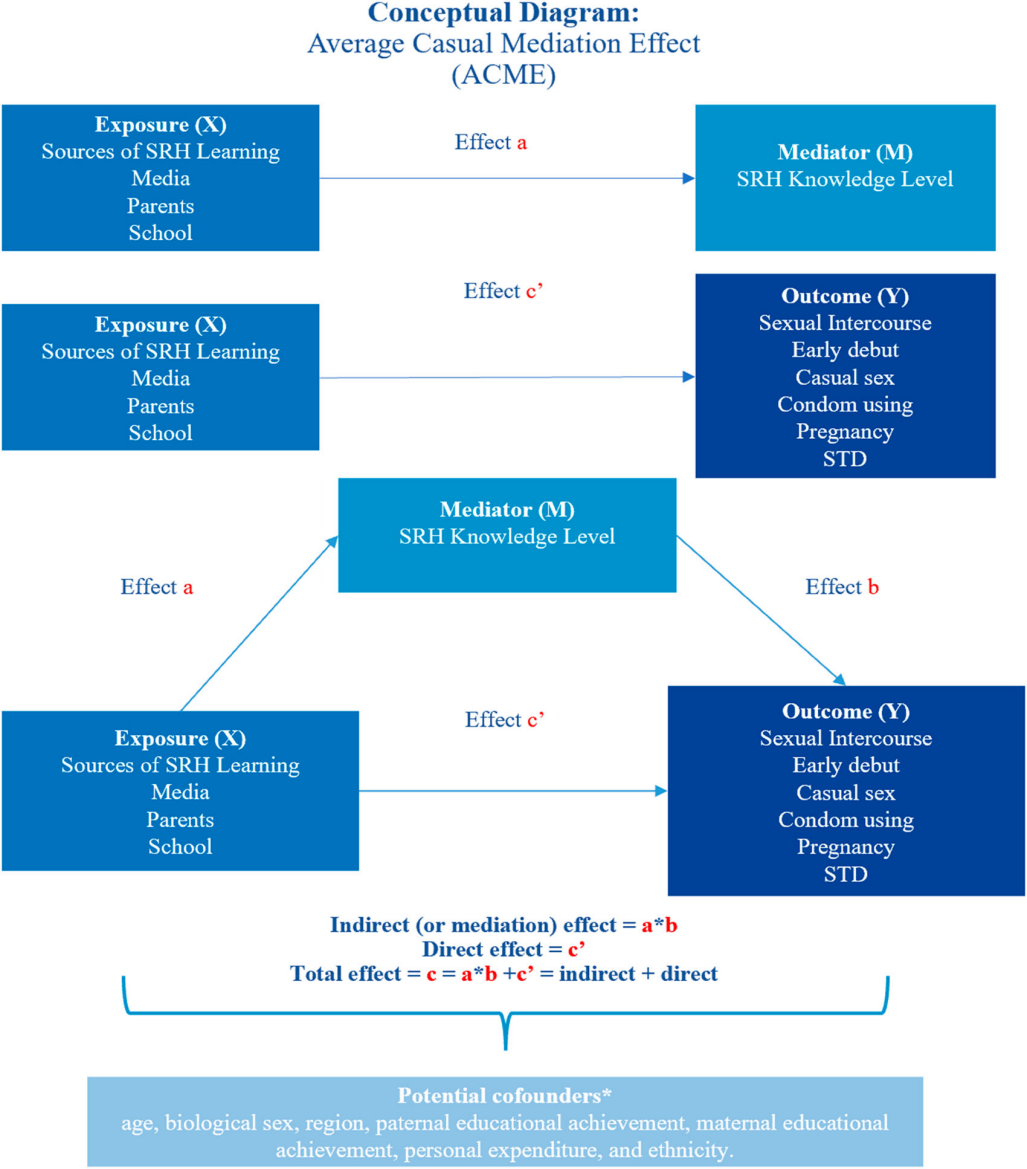
Hypothesis model

Fourth, the hypothesised mediating role of sexual knowledge scores (Path ab in Figure 1) was grounded in Basil Bernstein’s theoretical framework and tested using the mediation approach outlined by Imai et al.^37^ This method addresses the constraints of linear structural equation modelling (LSEM) and is suitable for nonlinear models. The mediation analysis included total effect (TE), average direct effect (ADE), average causal mediation effect (ACME), and proportion of mediation (PM), calculated with 1000 bootstrap resamples. Statistical analyses were conducted using R 4.1.0 with the mediation package^38^.

## Results

Of the 55,757 total samples collected, 1,177 (2%) were excluded due to incomplete attention check questions, or the informed consent. Then, 54,580 youth were included in the current study’s valid sample. Of these, 35,736 (65.5%) were female and 42,372 (77.6%) were heterosexual. According to the standard definition of the World Health Organisation (WHO), we defined youth as the group aged 15–24 years.^39^ The age-eligible participants were limited to youths aged 15–24 years, which excluded 995 participants. As the information on 987 participants’ paternal educational achievement and 342 participants’ maternal educational achievement was not available, they were excluded from our analysis. Finally, 52,256 participants were analysed in this study, including 25,873 (49.5%) heterosexual females, 8,531 (16.3%) sexual minority females, 14,865 (28.4%) heterosexual males, and 2,987 (5.7%) sexual minority males. Media use was more prevalent among males (15,639; 88.0%) than among females (23,644; 69.0%). Females reported higher rates of parental communication (9,060; 26.0%) and school education (20,301; 72.0%) compared to males (3,319; 19.0% and 9,294; 64.0%, respectively). Males reported higher rates of sexual intercourse (4,856; 27.0%), early debut (827; 4.6%), casual sex (1,111; 23.0%), and STIs (246; 1.4%) compared to females (6,547; 19.0%, 731; 2.1%, 842; 13.0%, and 324; 0.9%, respectively). Detailed characteristics of the participants are presented in Table 1.

**Table 1.** Characteristics of study participants by sexual orientation (*N* = 52,256)

Characteristic	Overall *N* = 52,256^a^	Female *N* = 34,404^a^	Male *N* = 17,852^a^	*P*-value^b^
*Sexual orientation*	* *	* *	* *	<0.001
Heterosexual	40,738 (78%)	25,873 (75%)	14,865 (83%)	
Minority	11,518 (22%)	8531 (25%)	2987 (17%)	
*Age group*				0.003
15–18	16,873 (32%)	11,098 (32%)	5775 (32%)	
19–20	25,188 (48%)	16,533 (48%)	8655 (48%)	
21–22	8035 (15%)	5406 (16%)	2629 (15%)	
23–24	2160 (4.1%)	1367 (4.0%)	793 (4.4%)	
*Region*				0.001
Rural	29,305 (56%)	19,471 (57%)	9834 (55%)	
Urban	22,951 (44%)	14,933 (43%)	8018 (45%)	
*Paternal educational achievement*				0.036
No formal schooling	1788 (3.4%)	1146 (3.3%)	642 (3.6%)	
Primary school	8510 (16%)	5701 (17%)	2809 (16%)	
Middle or high school	31,630 (61%)	20,807 (60%)	10,823 (61%)	
College and above	10,328 (20%)	6750 (20%)	3578 (20%)	
*Maternal educational achievement*	* *	* *	* *	0.500
No formal schooling	4119 (7.9%)	2698 (7.8%)	1421 (8.0%)	
Primary school	12,144 (23%)	8063 (23%)	4081 (23%)	
Middle or high school	27,813 (53%)	18,265 (53%)	9548 (53%)	
College and above	8180 (16%)	5378 (16%)	2802 (16%)	
*Personal expenditure per month(CNY)*	* *	* *	* *	<0.001
<1000	7786 (15%)	5293 (15%)	2493 (14%)	
1000–1499	17,962 (34%)	11,851 (34%)	6111 (34%)	
1500–2000	11,998 (23%)	7809 (23%)	4189 (23%)	
>2000	14,510 (28%)	9451 (27%)	5059 (28%)	
*Ethnicity*	* *	* *	* *	<0.001
Han	47,341 (91%)	30,974 (90%)	16,367 (92%)	
Minority	4915 (9.4%)	3430 (10.0%)	1485 (8.3%)	
*Media*				<0.001
No	12,973 (25%)	10,760 (31%)	2213 (12%)	
Yes	39,283 (75%)	23,644 (69%)	15,639 (88%)	
*Parental communication*	* *	* *	* *	<0.001
No	39,877 (76%)	25,344 (74%)	14533 (81%)	
Yes	12,379 (24%)	9060 (26%)	3319 (19%)	
*Sexual knowledge score*	4.00 (2.00, 6.00)	4.00 (2.00, 6.00)	4.00 (2.00, 6.00)	<0.001
*Sexual intercourse*	* *	* *	* *	<0.001
No	40,853 (78%)	27,857 (81%)	12,996 (73%)	
Yes	11,403 (22%)	6547 (19%)	4856 (27%)	
*Age at first intercourse*	* *	* *	* *	<0.001
<15	324 (2.8%)	146 (2.2%)	178 (3.7%)	
15–18	2688 (24%)	1305 (20%)	1383 (29%)	
18–20	5080 (45%)	2979 (46%)	2101 (43%)	
20–22	2503 (22%)	1585 (24%)	918 (19%)	
22–24	774 (6.8%)	519 (7.9%)	255 (5.3%)	
Not had sexual intercourse	40,887	27,870	13,017	
*Early debut*				<0.001
No	50,698 (97%)	33,673 (98%)	17,025 (95%)	
Yes	1558 (3.0%)	731 (2.1%)	827 (4.6%)	
*STIs*				<0.001
No	51,686 (99%)	34,080 (99%)	17,606 (99%)	
Yes	570 (1.1%)	324 (0.9%)	246 (1.4%)	
Casual sex*	* *	* *	* *	<0.001
No	9450 (83%)	5705 (87%)	3745 (77%)	
Yes	1953 (17%)	842 (13%)	1111 (23%)	
Not had sexual intercourse	40,853	27,857	12,996	
Condom use during the first intercourse*	* *	* *	* *	0.400
No	4100 (36%)	2377 (36%)	1723 (35%)	
Yes	7303 (64%)	4170 (64%)	3133 (65%)	
Not had sexual intercourse	40,853	27,857	12,996	
Pregnancy*	* *	* *	* *	*0*.*200*
No	10,938 (96%)	6293 (96%)	4645 (96%)	
Yes	465 (4.1%)	254 (3.9%)	211 (4.3%)	
Not had sexual intercourse	40,853	27,857	12,996	

* Analysis was in people who had sexual intercourse (*n* = 11,403).

a *n* (%); Median (IQR).

b Pearson's Chi-squared test; One-way ANOVA.

China Yuan (CNY); Sexually transmitted infections(STIs).

Associations between sources of sexual learning and sexual behaviours, and reproductive health outcome are presented in Table 2. Youths who learned from media were more likely to have sexual intercourse (OR 8.38, 95% CI 7.34–9.58), early debut (OR 8.91, 95% CI 6.21–12.79), STIs (OR 2.19, 95% CI 1.63–2.95), and casual sex (OR 3.38, 95% CI 1.83–6.23). For youths who had parental communication or learned from school, they were less likely to have sexual intercourse (parental communication: OR 0.82, 95% CI 0.77–0.87; school education: OR 0.75, 95% CI 0.71–0.80). However, there were no statistically significant associations between learning sources and condom use (media: OR 1.08, 95% CI 0.83–1.40; parental communication: OR 1.06, 95% CI 0.96–1.17; school education: OR 1.07, 95% CI 0.98–1.17), and pregnancy (media: OR 1.31, 95% CI 0.64–2.69; parental communication: OR 1.00, 95% CI 0.78–1.27; school education: OR 1.04, 95% CI 0.84–1.29).

**Table 2.** Multivariable logistic regression analysis of the association between learning sources and sexual behaviours

Characteristic	All participants *N* = 42,744			Participants who had sexual intercourse *N* = 9,506		
	Sexual intercourse	Early debut	STIs	Casual sex	Condom use	Pregnancy
	aOR (95% CI)	aOR (95% CI)	aOR (95% CI)	aOR (95% CI)	aOR (95% CI)	aOR (95% CI)
Sexual knowledge scores	1.27 (1.25, 1.29)***	1.14 (1.11, 1.17)***	0.94 (0.9, 0.98)**	0.99 (0.96, 1.02)	1.05 (1.03, 1.07)***	1.01 (0.95, 1.06)
*Media*						
No	Reference	Reference	Reference	Reference	Reference	Reference
Yes	8.38 (7.34, 9.58)***	8.91 (6.21, 12.79)***	2.19 (1.63, 2.95)***	3.38 (1.83, 6.23)***	1.08 (0.83, 1.40)	1.31 (0.64, 2.69)
*Parental communication*						
No	Reference	Reference	Reference	Reference	Reference	Reference
Yes	0.82 (0.77, 0.87)***	0.94 (0.83, 1.07)	1.33 (1.07, 1.64)**	1.15 (1.02, 1.3)*	1.06 (0.96, 1.17)	1.00 (0.78, 1.27)
*School Education*						
No	Reference	Reference	Reference	Reference	Reference	Reference
Yes	0.75 (0.71, 0.80)***	0.85 (0.76, 0.96)**	0.73 (0.60, 0.88)**	0.84 (0.75, 0.94)**	1.07 (0.98, 1.17)	1.04 (0.84, 1.29)
*Age group*						
15–18	Reference	Reference	Reference	Reference	Reference	Reference
19–20	1.66 (1.55, 1.78)***	0.71 (0.62, 0.81)***	1.15 (0.91, 1.47)	1.17 (0.98, 1.4)	1.17 (1.03, 1.32)*	0.94 (0.68, 1.29)
21–22	3.42 (3.15, 3.71)***	0.75 (0.64, 0.89)***	1.68 (1.27, 2.23)***	1.74 (1.44, 2.10)***	1.25 (1.09, 1.43)**	1.19 (0.85, 1.66)
23–24	5.28 (4.68, 5.95)***	0.52 (0.4, 0.69)***	2.57 (1.78, 3.71)***	1.96 (1.58, 2.44)***	1.21 (1.02, 1.43)*	1.41 (0.95, 2.08)
*Region*						
Rural	Reference	Reference	Reference	Reference	Reference	Reference
Urban	1.2 (1.13, 1.27)***	1.35 (1.19, 1.53)***	1.34 (1.09, 1.65)**	1.14 (1.01, 1.29)*	1.15 (1.05, 1.26)**	0.94 (0.75, 1.18)
*Maternal educational achievement*						
No formal schooling	Reference	Reference	Reference	Reference	Reference	Reference
Primary school	0.94 (0.83, 1.06)	1.04 (0.76, 1.42)	0.55 (0.39, 0.77)***	0.89 (0.66, 1.18)	1.33 (1.09, 1.63)**	0.70 (0.48, 1.03)
Middle or high school	1.03 (0.91, 1.15)	1.3 (0.97, 1.75)	0.49 (0.36, 0.68)***	0.99 (0.76, 1.30)	1.43 (1.18, 1.73)***	0.48 (0.33, 0.69)***
College and above	0.98 (0.85, 1.13)	1.19 (0.85, 1.66)	0.58 (0.37, 0.89)***	0.96 (0.70, 1.30)	1.49 (1.19, 1.87)***	0.48 (0.29, 0.79)**
*Paternal educational achievement*						
No formal schooling	Reference	Reference	Reference	Reference	Reference	Reference
Primary school	0.9929 (0.8341, 1.1819)	0.96 (0.65, 1.42)	0.42 (0.29, 0.63)***	0.91 (0.63, 1.32)	0.9 (0.68, 1.2)	0.95 (0.56, 1.6)
Middle or high school	0.98 (0.83, 1.16)	0.83 (0.57, 1.2)	0.37 (0.26, 0.52)***	0.74 (0.52, 1.05)	1.05 (0.8, 1.37)	0.7 (0.42, 1.16)
College and above	0.83 (0.7, 0.99)*	0.67 (0.45, 1)	0.33 (0.21, 0.51)***	0.77 (0.53, 1.12)	1.17 (0.88, 1.57)	0.55 (0.31, 0.99)*
*Household outcome (CNY)*						
<1000	Reference	Reference	Reference	Reference	Reference	Reference
1000–1499	1.37 (1.23, 1.53)***	0.99 (0.76, 1.29)	0.72 (0.54, 0.97)*	1.02 (0.73, 1.43)	1.21 (0.99, 1.48)	1.09 (0.7, 1.71)
1500–2000	2.13 (1.91, 2.38)***	1.65 (1.27, 2.13)***	0.75 (0.55, 1.03)	1.36 (0.98, 1.88)	1.33 (1.09, 1.63)**	0.65 (0.41, 1.05)
>2000	4.18 (3.75, 4.65)***	3.32 (2.6, 4.25)***	1.1 (0.81, 1.48)	2.4 (1.76, 3.27)***	1.25 (1.03, 1.51)*	1.09 (0.7, 1.69)
*Ethnicity*						
Han	Reference	Reference	Reference	Reference	Reference	Reference
Minority	1.18 (1.08, 1.29)***	1.37 (1.14, 1.65)**	1.21 (0.91, 1.59)	0.98 (0.81, 1.19)	0.69 (0.6, 0.79)***	1.19 (0.87, 1.62)

All models adjusted for knowledge scores, media, maternal communication, paternal communication, school education, age, region, personal expenditure, paternal educational achievement, maternal educational achievement, and ethnicity.

*p < 0.05, **p < 0.01, ***p < 0.001.

Adjusted odds ratio (aOR); confidence interval (CI); reference (Ref); China Yuan (CNY); Sexually transmitted infections (STIs).

**Figure 2.**
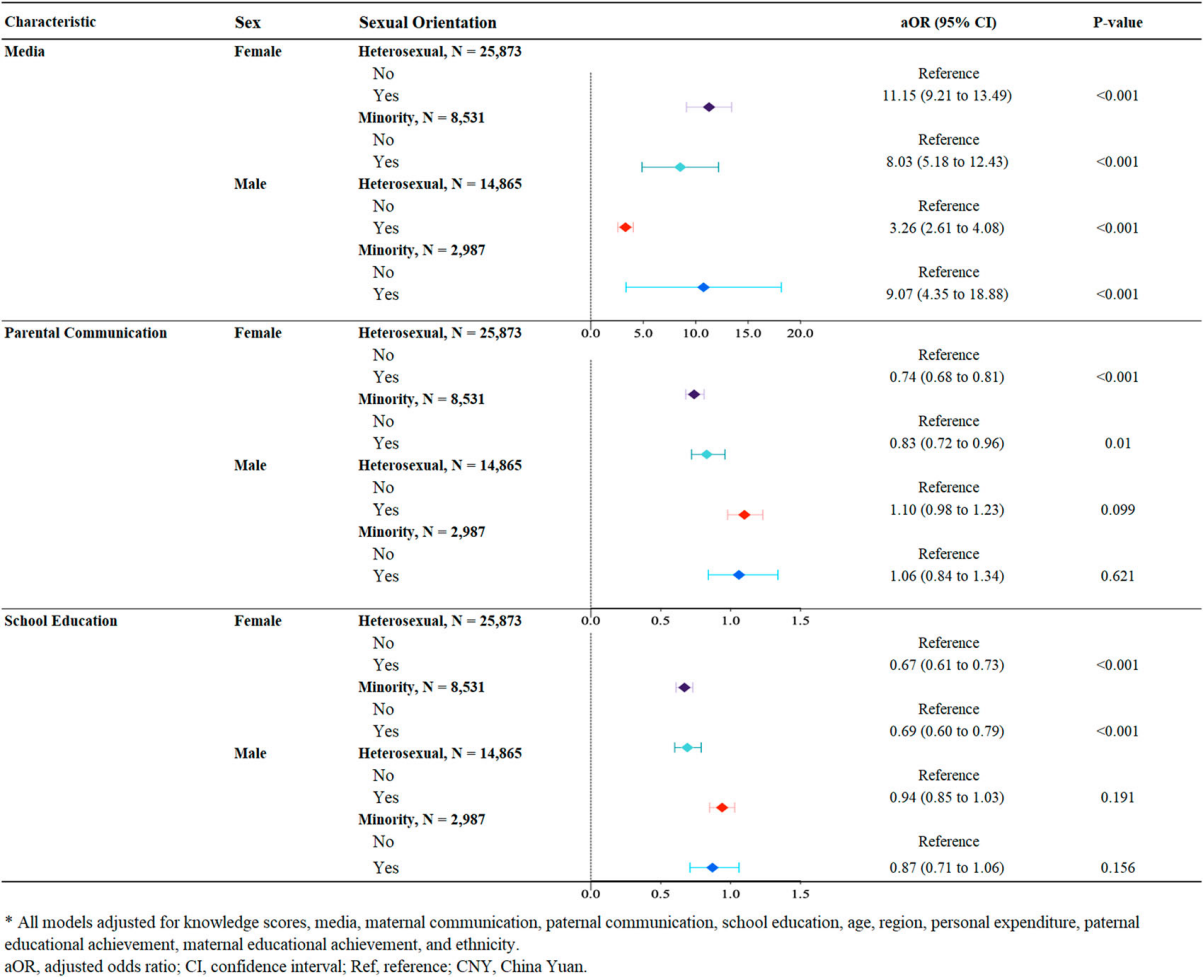
Multivariable logistic regression analysis of the association between learning sources and sexual intercourse, stratified by sexual orientation groups, sexual intercourse, stratified by sexual orientation groups

Associations between sources of sexual learning and sexual intercourse are presented in Figure 2. Youths who learned from media, regardless of sexual orientation, were more likely to have sexual intercourse (heterosexual females: OR 11.15, 95% CI 9.21–13.49, minority females: OR 8.03, 95% CI 5.18–12.43, heterosexual males: OR 3.26, 95% CI 2.61–4.08, heterosexual males: OR 9.07, 95% CI 4.35–18.88). Of youths who had parental communication or learned from school, regardless of sexual orientation, females were less likely to have sexual intercourse (parental communication: OR 0.74, 95% CI 0.68–0.81 for heterosexual females, OR 0.83, 95% CI 0.72–0.96 for minority females; school education: OR 0.67, 95% CI 0.61–0.73 for heterosexual females, OR 0.69, 95% CI 0.60–0.79 for minority females).

Table 3 presents the TE, ADE, ACME (mediated by sexual knowledge scores), and PM of the association between sexual learning sources and sexual behaviours and reproductive health outcomes. The estimated coefficients in the table can be interpreted as the increased/decreased probability of sexual behaviours and reproductive health outcomes, comparing youth who learned from sexual learning sources with those who did not. Sexual knowledge score played a positive mediation role in the association between media and sexual intercourse (PM 12.59, 95% CI 11.76–13.00), media and early debut (PM 8.92, 95% CI 7.29–11.00). For parental communication, no mediating effect was observed. For school education, there is a partly negative mediated effect of sexual knowledge scores between media and sexual intercourse (PM −54.96%, 95% CI −75.88 to −41.00), and also a negative mediated effect of sexual knowledge scores between media and early debut (PM −60.46%, 95% CI −249.09 to −23.00).

**Table 3.** The mediating effect of sexual knowledge scores in the association between learning source and multiple sexual behaviours

Characteristic	All participants *N* = 42,744			Participants who had sexual intercourse *N* = 9,506		
	Sexual intercourse	Early debut	STD	Casual sex	Condom use	Pregnancy
	Coef./proportion (95% CI)	Coef./proportion (95% CI)	Coef./proportion (95% CI)	Coef./proportion (95% CI)	Coef./proportion (95% CI)	Coef./proportion (95% CI)
Media						
ACME	0.03 (0.03–0.03)***	0.003 (0.002–0.003)***	−0.0005 (−0.0010 to – 0.0002)*	−0.003 (−0.007–0.001)	0.016 (0.010 to o.020)***	0.001 (−0.001–0.002)
ADE	0.19 (0.18–0.20)***	0.030 (0.028–0.030)***	0.007 (0.005–0.010)***	0.100 (0.063–0.130)***	0026 (−0.032–0.080)	0.000 (−0.028–0.20)
TE	0.22 (0.21–0.22)***	0.033 (0.031–0.040)***	0.006 (0.005–0.010)***	0.097 (0.059–0.130)***	0.042 (−0.015–0.100)	0.001 (−0.025–0.020)
PM(%)	12.59 (11.76–13.00)***	8.92 (7.29–11.00)***	−7.96 (−16.41 to – 1.00)*	–	–	–
*Parental communication*						
ACME	0.02 (0.02–0.03)***	0.003 (0.003–0.003)***	−0.00 (−0.00–0.00)	−0.00 (−0.00–0.00)	0.004 (0.002–0.010)***	0.00 (−0.00–0.00)
ADE	−0.02 (−0.03 to – 0.01)***	−0.002 (−0.005–0.002)	0.003 (0.001–0.010)*	0.011 (−0.006–0.030)	0.011 (−0.008–0.030)	−0.002 (−0.010–0.010)
TE	0.004 (−0.003–0.010)	0.001 (−0.002–0.003)	0.003 (0.000–0.010)*	0.010 (−0.007–0.030)	0.015 (−0.004–0.040)	−0.001 (−0.010–0.010)
PM(%)	–	–	–	–	–	–
*School education*						
ACME	0.016 (0.014–0.020)***	0.0021 (0.0017–0.0025)***	−0.00 (−0.00–0.00)	0.00 (−0.0005–0.00)	0.0015 (0.0004–0.002)**	0.00 (−0.00–0.00)
ADE	−0.045 (−0.053 to – 0.040)***	−0.005 (−0.009 to – 0.001)**	−0.003 (−0.006 to – 0.001)***	−0.024 (−0.039 to – 0.010)**	0.015 (−0.004–0.040)	0.00 (−0.00–0.01)
TE	−0.029 (−0.038 to – 0.020)***	−0.003 (−0.007–0.00)*	−0.004 (−0.006 to – 0.002)***	−0.024 (−0.039 to – 0.010)**	0.017 (−0.003–0.040)	0.00 (−0.00–0.01)
PM(%)	−54.96 (−75.88 to – 41.00)***	−60.46 (−249.09 to – 23.00)*	–	–	–	–

All models adjusted for age, region, personal expenditure, paternal educational achievement, maternal educational achievement, and ethnicity.

*p < 0.05, **p < 0.01, ***p < 0.001.

Average causal mediation effect (ACME); average direct effect (ADE); proportion of mediation (PM); total effect (TE); coefficient (Coef.); Sexually transmitted infections(STIs).

For youths who learned from the media, regardless of sexual orientation, the results showed ACME of sexual knowledge scores and ADE, indicating a partly mediated effect of sexual knowledge scores (see Supplemental Tables S2 and S3). For sexual intercourse, the PM of sexual knowledge scores were higher in sexual minority females (PM 22.35, 95%CI 19.01–26.00) than in heterosexual males (PM 19.61, 95%CI 16.59–23.00), heterosexual females (PM 15.37, 95%CI 14.15–17.00), and sexual minority males (PM 14.69, 95%CI 10.72–20.00) (see Supplemental Table S2). For early debut, the PM of sexual knowledge scores were higher in sexual minority females (PM 16.56, 95%CI 11.18–24.00), than in sexual minority males (PM 16.30, 95% CI 7.09–33.00), heterosexual females (PM 13.23, 95% CI 10.38–16.00), and heterosexual males (PM 12.73, 95% CI 7.93–21.00) (see Supplemental Table S3).

For youths who learned from parental communication, no mediating effect was observed between parental communication and sexual intercourse (see Supplemental Table S2). There is a partly mediated effect of sexual knowledge scores between parental communication and early debut in heterosexual males (PM 29.20%, 95% CI 12.58–186.00) and sexual minority males (PM 13.78%, 95% CI 5.44–41.00) (see Supplemental Table S3). For youths who learned from school education, there is a partly mediated effect of sexual knowledge scores between school education and sexual intercourse in heterosexual females (PM −51.70%, 95% CI −81.26 to −36.00) and sexual minority females (PM −45.80%, 95% CI −111.65 to −26.00) (see Supplemental Table S2).

## Discussion

Our national study in China shows how different patterns of SRH knowledge are associated with youth sexual behaviours, demonstrating several country-specific findings.

First, our results show that youths’ SRH knowledge learning patterns and sexual behaviours are gendered.^40^ For instance, young males are more likely than young females to use media as an information source, whereas females are more likely to obtain knowledge from school-based sexuality education and parental communications. It should be noted that in this study, media-based learning was primarily driven by exposure to pornographic materials rather than searching for SRH knowledge (e.g. contraception) online. Apart from that, regardless of sexual orientation, more young males in our study had had sexual intercourse and an earlier sexual debut (<16 years old), casual sex, and adverse SRH outcomes (i.e. STIs) than young females. Gender differences in sexual behaviours have also been shown in existing China studies over the last two decades^41,42^. Although people’s attitudes toward premarital sex have become more open, China’s patriarchal Confucian tradition still expects women to retain premarital chastity and be less sexually active than men.^43,44^ Premarital sex for girls is often stigmatised. For example, a large-scale local study in 2016 reported that both female and male participants believe that premarital sex among females is less acceptable than among males.^45^ Such a double standard gives males more permission than females to engage in sexually related exploration, while emphasising the need for females to bear the consequences, reinforcing stereotypical norms and gender inequalities in sexual behaviour.^21^

Second, youths who learnt SRH knowledge through parental communications showed lower possibilities of having sexual intercourse than their counterparts. This is consistent with previous findings that parent–adolescent communication serves as a protective factor for adolescents, leading to a delay in their sexual initiation, as well as safer sexual behaviours.^46,47^ More specific analyses demonstrate that parental communication provides more robust protection for girls than boys regarding safe sexual behaviours.^48,49^ However, when the mediating effect of sexual knowledge scores was applied and stratified by gender, opposite results emerged. Young males’ communication with their parents increased their sexual knowledge scores, but also encouraged their early sexual debut. This finding has not been shown in Chinese young females. Such a phenomenon could be due to gender-biased parental communication and double standards, such as the expectations for young females to maintain premarital chastity discussed above. Meanwhile, a qualitative study in China suggests that parents tend to emphasise the negative consequences of having a romantic relationship and the prohibition of having sexual intercourse with daughters rather than sons.^21^ Such gender differences have also been found in other countries, reporting daughters as the main focus of family communication about sexually related topics^50^.

Third, compared to gaining SRH information from parents as an informal learning source, learning from the media is more sex-positive and permissive.^51^ Our results show that youths who had been exposed to sexual information on social and other media (mainly pornographic materials) were more likely to have sexual intercourse, early sexual debut, casual sex, and STIs than those who had not. This finding supports the social ecology theory that mass media are a key dimension of young people’s lives and are closely linked to risky sexual behaviour among adolescents.^52^ After conducting the mediation analysis, the results show that regardless of gender or sexual orientation, youths’ sexual knowledge score increases through media learning (mainly pornographic materials), leading to higher possibilities of having sexual intercourse and early sexual debut. This finding resonates with previous studies that demonstrate a positive association between pornography use and (debuting) sexual intercouse.^35,53^ For instance, this association could be interpreted as suggesting young people’s primary motivation for using pornography is to gain sexual pleasure or excitement.^28,54^ However, it has also been suggested that, especially in Asian countries where parents and educators hesitate about teaching sex, media serve as a “super peer” for young people to acquire sexual knowledge less embarrassingly.^43^ Studies further argue that adolescents who learn sexual knowledge from the media are more likely to adopt “observational learning” or “behavioural modelling”.^26^ The negative consequences of sexual activities are less likely to appear in the media, especially in pornography, leading to increased curiosity about sexual behaviour^43^.

Fourth, in contrast to parental communication and media as informal channels, school-based sexuality education is a formal way for youths to acquire positive and accurate SRH knowledge.^17^ In our results, youths who have had formal school-based sexuality education showed lower possibilities than their counterparts who had not, of having sexual intercourse, early sexual debut, casual sex, and adverse SRH outcomes. However, after examining the gender difference in the mediation analysis, only young females who had higher SRH knowledge scores from school-based learning showed a lower likelihood of sexual intercourse. This indicates that formal sexuality education at school potentially protects young females by delaying sexual debut.^55^ However, this result is partially inconsistent with the international guidance on sexuality education, which demonstrates that effective sexuality education could delay the initiation of sexual intercourse in both young females and males.^4^ A study in China found a positive association between school-based sexuality education and the likelihood of sexual initiation among young males, suggesting that the “good intention” of such education does not necessarily delay first sexual intercourse^56^.

It should be noted that an understudied cultural phenomenon may have some bearing on our findings, that is, the virgin shaming of young males which is part of the sexual double standard. A US study in 2018 pointed out such a phenomenon, suggesting that masculine behaviours tend to reproduce themselves and suppress images of masculinity that do not conform to social or cultural norms, leading to discrimination against virginity among males imposed by other men and even male virgins themselves.^57^ Therefore, if Chinese youth cannot receive quality school-based sexuality education that promotes SRH and gender and sexuality equality, young Chinese males could potentially experience virgin shaming and thus intend to initiate sexual intercourse.

### Implications and limitations

The 2030 Agenda for Sustainable Development indicates that quality education, good health and well-being, and gender equality are inherently interconnected.^4^ Therefore, creating positive SRH learning sources is important for empowering youths’ lives and future. This study has several implications. First, the credibility and trustworthiness of SRH learning sources plays a role in adolescents’ sexual behaviour.^26^ This indicates that adolescents place more weight on SRH knowledge gained from parental communication and school-based sexuality education than from the media. Therefore, parents in China need additional training to enter into practical, unbiased, and quality sexual communication with their children, emphasising the joint responsibility of all genders regarding SRH. Simultaneously, a curriculum-based comprehensive sexuality education that teaches children and young people about the cognitive, emotional, physical and social aspects of sexuality should be covered in China. Educators should be formally trained. Gender equality should also be promoted to overcome cultural phenomena such as virgin shaming to achieve a more inclusive and gender-equal environment. In addition, the government needs to create a rating system according to sexual, violent or discriminatory content, for movies, videos, and other mass media in order to protect minors. Interventions from professionals, schools, and parents are also needed to help youths understand and critically process received information to avoid potential risks^30^.

A fundamental limitation of this study is its cross-sectional design, which means we cannot assume that the associations we found are causal. Also, the media learning in the survey design emphasises the consumption of pornographic materials, which do not comprehensively represent SRH knowledge learning from mass media. Meanwhile, peer learning was not included as an informal learning source. Future research exploring this topic could consider peer SRH knowledge learning experiences. Moreover, the data were self-reported, and the relatively conservative social norms regarding sex may lead to underreporting of sexual intercourse. Additionally, the study findings are not representative of and cannot be generalised to all Chinese youth since the study was limited to college students. Our use of snowball sampling effectively recruited a large number of participants but introduced potential selection bias and limited the generalisability of findings. The sampling method may overrepresent students who were willing to discuss sexual and reproductive health topics or who had stronger connections to institutional networks. Future studies could benefit from employing probability sampling methods to achieve more representative samples of Chinese youth.

## Conclusion

Underpinned by Bernstein’s theoretical framework of formal and informal learning, this study’s findings reveal the association of both formal and informal SRH knowledge learning and sexual behaviour, with a model to test the hypothesised mediating role of sexual knowledge scores. The results highlight how different settings of SRH knowledge learning affect knowledge acquisition and sexual behaviour among youth, demonstrating gender differences. This study contributes to the literature on Chinese youth’s SRH learning patterns and sexual behaviour. It also provides implications for improving SRH learning sources in different settings to support the transition of children and young people to safe and healthy adulthood.

## Supplementary Material

Supplemental Material
