# Supplementary material for: Association between Chinese youth’s sources of sexual knowledge and sexual and reproductive health: a mediation analysis of sexual knowledge level
Source: Sex Reprod Health Matters. 2025 Aug 4;33(1):2517430. doi: 10.1080/26410397.2025.2517430 (PMC12337732; doi:10.1080/26410397.2025.2517430)
Supplement: Supplemental Material [file ZRHM_A_2517430_SM8077.docx]

**Supplemental table 1** **Questions to assess SRH knowledge**

| **Dimension** |  | **Items (Participants could answer: True, False, or Unknown)** |
| --- | --- | --- |
| 1. Contraceptive Use | | Condoms are the only types of contraception that protect against STIs, and pregnancy |
|  |  | A woman is more likely to get pregnant if she has sexual intercourse around 14 days before menstruation |
| 2. Abortion/Pregnancy | | When sperm are inside women's body, they can stay alive for about 7 days |
|  |  | Withdrawal is an effective way to prevent pregnancy |
|  |  | Having sex during safe period is an effective way to prevent pregnancy. |
|  |  | If a woman gets pregnant, her menstrual periods would continue for two or three months. |
|  |  | Painless surgical abortion is safer than a regular surgical abortion. |
| 3. STIs |  | Mosquito's bite can transmit HIV/AIDS |
|  |  | Genital herpes is a sexually transmitted infection |

SRH: sexual and reproductive health; STIs: sexually transmitted infections

**Supplemental table 2 The Mediating Effect of Sexual Knowledge Scores in the association between learning source and sexual intercourse, stratified by sex and sexual orientation**

| **Characteristic** |  | **Female** | |  | **Male** | |
| --- | --- | --- | --- | --- | --- | --- |
|  |  | **Heterosexual** | **Minority** |  | **Heterosexual** | **Minority** |
|  |  | **N = 25,873** | **N = 8,531** |  | **N = 14,865** | **N = 2,987** |
|  |  | **Coef./proportion (95%CI)** | **Coef./proportion (95%CI)** |  | **Coef./proportion (95%CI)** | **Coef./proportion (95%CI)** |
| **Media** |  |  |  |  |  |  |
| ACME |  | 0.03 (0.03 to 0.03)*** | 0.04 (0.04 to 0.05)*** |  | 0.03 (0.03 to 0.04)*** | 0.04 (0.03 to 0.06)*** |
| ADE |  | 0.17 (0.17 to 0.18)*** | 0.15 (0.14 to 0.17)*** |  | 0.14 (0.12 to 0.16)*** | 0.25 (0.20 to 0.29)*** |
| TE |  | 0.21 (0.20 to 0.21)*** | 0.20 (0.18 to 0.21)*** |  | 0.18 (0.16 to 0.19)*** | 0.29 (0.24 to 0.32)*** |
| PM(%) |  | 15.37 (14.15 to 17.00)*** | 22.35 (19.01 to 26.00)*** |  | 19.61 (16.59 to 23.00)*** | 14.69 (10.72 to 20.00)*** |
| **Parental communication** | | |  |  |  |  |
| ACME |  | 0.02 (0.02 to 0.03)*** | 0.03 (0.02 to 0.03)*** |  | 0.02 (0.02 to 0.03)*** | 0.02 (0.01 to 0.03)*** |
| ADE |  | -0.02 (-0.03 to -0.01)*** | -0.02 (-0.04 to 0.00) |  | 0.02 (0.00 to 0.03) | 0.02 (-0.02 to 0.05) |
| TE |  | 0.00 (-0.01 to 0.01) | 0.00 (-0.01 to 0.02) |  | 0.04 (0.02 to 0.06)*** | 0.03 (-0.00 to 0.07) |
| PM(%) |  | - | - |  | - | - |
| **School Education** | | |  |  |  |  |
| ACME |  | -0.03 (-0.05 to -0.02)*** | -0.04 (-0.06 to -0.02)*** |  | 0.00 (-0.01 to 0.02) | -0.02 (-0.05 to 0.02) |
| ADE |  | 0.02 (0.02 to 0.02)*** | 0.02 (0.01 to 0.02)*** |  | 0.01 (0.01 to 0.02)*** | 0.01 (0.00 to 0.02)*** |
| TE |  | -0.05 (-0.06 to -0.04)*** | -0.06 (-0.08 to -0.04)*** |  | -0.01 (-0.03 to 0.00) | -0.03 (-0.06 to 0.01) |
| PM(%) |  | -51.70 (-81.26 to -36.00)*** | -45.80 (-111.65 to -26.00)*** |  | - | - |

All models adjusted for age, region, personal expenditure, paternal educational achievement, maternal educational achievement, and ethnicity.

*p<0.05, **p<0.01, ***p<0.001.

ACME, average causal mediation effect; ADE, average direct effect; PM, proportion of mediation; TE, total effect; Coef., coefficient.

**Supplemental table 3 The Mediating Effect of Sexual Knowledge Scores in the association between learning source and early debut, stratified by sex and sexual orientation**

|  |  | **Early debut** | | | | |
| --- | --- | --- | --- | --- | --- | --- |
| **Characteristic** |  | **Female** | |  | **Male** | |
|  |  | **Heterosexual** | **Minority** |  | **Heterosexual** | **Minority** |
|  |  | **N = 25,873** | **N = 8,531** |  | **N = 14,865** | **N = 2,987** |
|  |  | **Coef./proportion (95%CI)** | **Coef./proportion (95%CI)** |  | **Coef./proportion (95%CI)** | **Coef./proportion (95%CI)** |
| **Media** |  |  |  |  |  |  |
| ACME |  | 0.003 (0.003 to 0.003)*** | 0.006 (0.004 to 0.010)*** |  | 0.004 (0.003 to 0.05)*** | 0.010 (0.005 to 0.020)*** |
| ADE |  | 0.021 (0.018 to 0.020)*** | 0.028 (0.023 to 0.030)*** |  | 0.026 (0.018 to 0.030)*** | 0.052 (0.029 to 0.070)*** |
| TE |  | 0.024 (0.022 to 0.030)*** | 0.034 (0.028 to 0.040)*** |  | 0.030 (0.023 to 0.040)*** | 0.062 (0.042 to 0.080)*** |
| PM(%) |  | 13.23 (10.38 to 16.00)*** | 16.56 (11.18 to 24.00)*** |  | 12.73 (7.93 to 21.00)*** | 16.30 (7.09 to 33.00)*** |
| **Parental communication** | | |  |  |  |  |
| ACME |  | 0.003 (0.002 to 0.004)*** | 0.004 (0.003 to 0.005)*** |  | 0.003 (0.002 to 0.004)*** | 0.005 (0.002 to 0.010)*** |
| ADE |  | -0.001 (-0.005 to 0.003) | -0.009 (-0.017 to -0.001)* |  | 0.007 (-0.002 to 0.020) | 0.032 (0.008 to 0.060) ** |
| TE |  | 0.002 (-0.002 to 0.010) | -0.005 (-0.012 to 0.002) |  | 0.009 (0.001 to 0.020)* | 0.037 (0.014 to 0.060)*** |
| PM(%) |  | - | - |  | 29.20 (12.58 to 186.00)* | 13.78 (5.44 to 41.00)*** |
| **School Education** | |  |  |  |  |  |
| ACME |  | 0.002 (0.002 to 0.002)*** | 0.003 (0.002 to 0.004)*** |  | 0.001 (0.001 to 0.001)*** | 0.028 (0.001 to 0.010)** |
| ADE |  | -0.005 (-0.009 to -0.001)* | -0.010 (-0.019 to -0.001)* |  | 0.003 (-0.004 to 0.010) | -0.014 (-0.036 to 0.008) |
| TE |  | -0.003 (-0.007 to 0.001) | -0.007 (-0.016 to 0.002) |  | 0.005 (-0.002 to 0.010) | -0.011 (-0.032 to 0.010) |
| PM(%) |  | - | - |  | - | - |

All models adjusted for age, region, personal expenditure, paternal educational achievement, maternal educational achievement, and ethnicity.

*p<0.05, **p<0.01, ***p<0.001.

ACME, average causal mediation effect; ADE, average direct effect; PM, proportion of mediation; TE, total effect; Coef., coefficient.
